# Rationale and design of the HERZCHECK trial: Detection of early heart failure using telemedicine and cardiovascular magnetic resonance in structurally weak regions (NCT05122793)

**DOI:** 10.1016/j.jocmr.2025.101841

**Published:** 2025-01-15

**Authors:** Sebastian Kelle, Anna Clara Nolden, Maximilian Leo Müller, Rebecca Elisabeth Beyer, Henning Steen, Bjoern Andrew Remppis, Johannes Wieditz, Hannah Kentenich, Alex Tuit, Mina Cvetkovic, Undine Ella Witt, Florian André, Sein Schmidt, Alexander Huppertz, Dusan Simic, Dirk Müller, Arim Shukri, Matthias Issing, Andre Glardon, Katrin Christiane Reber, Ulf Landmesser, Norbert Frey, Burkert Pieske, Stephanie Stock, Volkmar Falk, Tim Friede, Gisela Thiede

**Affiliations:** a Department of Cardiology, Angiology and Intensive Care Medicine, Deutsches Herzzentrum der Charité, Berlin, Germany; b Charité - Universitätsmedizin Berlin, Berlin, Germany; c DZHK (German Centre for Cardiovascular Research), Partner Site Berlin, Berlin, Germany; d Department of Cardiology, Angiology and Pneumology, University Hospital Heidelberg, Heidelberg, Germany; e DZHK (German Centre for Cardiovascular Research), Partner Site Heidelberg, Heidelberg, Germany; f Heart and Vascular Center Bad Bevensen, Bad Bevensen, Germany; g Department of Medical Statistics, University Medical Center Göttingen, Göttingen, Germany; h Institute for Health Economics and Clinical Epidemiology (IGKE), Faculty of Medicine and University Hospital Cologne, University of Cologne, Cologne, Germany; i Herzinstitut Berlin, Kardiologische Gemeinschaftspraxis, Berlin, Germany; j Berlin Institute of Health (BIH) at Charité – Universitätsmedizin Berlin, Berlin, Germany; k Department of Neurology, Charité- Universitätsmedizin Berlin, Berlin, Germany; l Center for Stroke Research (CSB), Charité- Universitätsmedizin Berlin, Berlin, Germany; m University of Potsdam, University Outpatient Clinic, Sports Medicine and Sports Orthopaedics, Potsdam, Germany; n medneo, Berlin, Germany; o roclub, Berlin, Germany; p AOK Nordost. Die Gesundheitskasse, Health Services Management, Berlin, Germany; q Department of Cardiology, Angiology, and Intensive Care, Deutsches Herzzentrum der Charité, Campus Benjamin Franklin, Berlin, Germany; r Division of Cardiology, University Medicine Rostock, Rostock, Germany; s Department of Cardiothoracic and Vascular Surgery, Deutsches Herzzentrum der Charité (DHZC), Berlin, Germany; t DZHK (German Centre for Cardiovascular Research), Partner Site Lower Saxony, Göttingen, Germany

**Keywords:** Stage B heart failure, Pre-heart failure, Screening, Prevention, Cardiac magnetic resonance imaging, Randomized controlled trial

## Abstract

**Background and aims:**

Heart failure (HF) is an imminent global health problem. Yet established screening algorithms for asymptomatic pre-HF, allowing for early and effective preventive interventions, are largely lacking. The HERZCHECK trial, conducted in structurally underserved rural regions of North-Eastern Germany, aims to close this gap by evaluating the feasibility, diagnostic efficacy, and cost-effectiveness of a fully mobile, telemedically-supervised screening approach, combining cardiovascular magnetic resonance (CMR) imaging and laboratory testing as central elements.

**Study Design and Methodology:**

The HERZCHECK trial is a prospective, randomized controlled trial employing a prospective randomized open blinded endpoint design. The study targets asymptomatic adults aged 40–69 years without a history of HF, but with at least one of the following cardiovascular risk factors: hypertension, hypercholesterolemia, obesity, smoking/tobacco consumption, chronic diabetes mellitus, or chronic kidney disease. Participants undergo a comprehensive screening examination including a questionnaire-based medical history, laboratory testing, and CMR at baseline. Based on CMR-derived global longitudinal strain (GLS), participants are classified as stratum A (GLS < −15%), B (GLS ≥ −15% to < −11%), or C (GLS ≥ −11%), with strata B and C being defined as asymptomatic pre-HF. Ten percent of participants in stratum A and all of stratum B and C are subsequently randomized into two groups, receiving either conventional or innovative medical reports, the latter including information on GLS, guideline-based recommendations, and access to a lifestyle intervention app for cardiovascular prevention. Additionally, treating physicians of participants in the innovative group are granted access to an expert center for telemedical inquiries. Follow-up assessments are performed over 12 months to evaluate changes in GLS, as well as adverse cardiac events and quality of life.

**Conclusion:**

HERZCHECK aims to provide a blueprint for a comprehensive, contemporary screening approach tailored to the needs of the targeted structurally underserved population. By implementing this approach in a representative at-risk cohort, HERZCHECK will provide important new information about (a) the prevalence of asymptomatic pre-HF in at-risk patients and (b) the feasibility, added diagnostic value and health economic aspects of CMR exams as part of future screening mechanisms for HF in clinical routine care (NCT05122793).

## 1 Background and Aims

With 1–2% of the adult population (i.e., >60 million people worldwide) already affected and a prevalence that is assumed to rise, heart failure (HF) is considered as a global epidemic with far-reaching consequences [1], [2], [3]. Beyond severe reductions in the quality of life (QoL) and life expectancy of individual patients, these comprise major socioeconomic implications [1], [2], [4], [5]. Specifically, it is estimated that HF causes an annual health care expenditure of more than $300 billion (i.e., >$5000 per patient) worldwide, with the majority attributable to repeated and protracted hospitalizations (approximately 1 per patient/year) [1], [2], [4], [5]. Thus, effective strategies to reduce the personal and socioeconomic burden of HF are much sought after.

One strategy that has proven effective in other diseases is a structured diagnostic routine that screens for early pre-symptomatic disease stages, corresponding to patients at risk of HF (stage A HF) and with asymptomatic pre-HF (stage B HF), the latter being characterized by structural cardiac abnormalities as defined in current HF guidelines by the American Heart Association (AHA) [6]. In particular, early identification of asymptomatic pre-HF (stage B HF) among the overall population at-risk (stage A HF) will facilitate targeted risk factor modifications, lifestyle adaptations, or pharmacotherapeutic interventions which may halt or postpone the transition to symptomatic HF (stages C and D HF) [6]. The potential efficacy of this approach has previously been demonstrated for a natriuretic peptide biomarker-based screening, which can be useful in patients with stage A HF according to the 2022 AHA HF guidelines [6], [7], [8]. However, a more holistic diagnostic workup, which incorporates the assessment of structural heart disease and increased filling pressures—the two other central aspects of asymptomatic pre-HF (stage B HF)—is required to exploit the full potential of screening for asymptomatic pre-HF (stage B HF) [6].

Apart from the current focus on natriuretic peptides, the lack of access to specialized health care is another important factor impeding the detection of asymptomatic pre-HF (stage B HF) in clinical routine care. This aspect is particularly relevant in structurally weak rural regions which suffer from an even higher prevalence of HF than urban regions but where the number and density of cardiologists is low, and services from general practitioners are declining [9], [10].

The central aim of the HERZCHECK trial is to overcome these problems by comprehensively evaluating a fully mobile, telemedically-supervised, and holistic screening routine, incorporating cardiac magnetic resonance imaging (CMR) and laboratory testing as central diagnostic methods.

## 2 Study design and methodology

### 2.1 Overview of study design

HERZCHECK is a prospective, randomized controlled trial with stratification and blinded assessment of the endpoint (prospective randomized open blinded endpoint [PROBE] design) that was initiated and planned by a consortium led by the Deutsches Herzzentrum der Charité. A list of all consortium members and technical partners is provided in Supplementary Table S1. The study is fully funded by the “Innovation Fund for the promotion of new forms of care” of the Federal Joint Committee (Gemeinsamer Bundesausschuss) and carried out between October 2020 and September 2024.

**Fig. 1:**
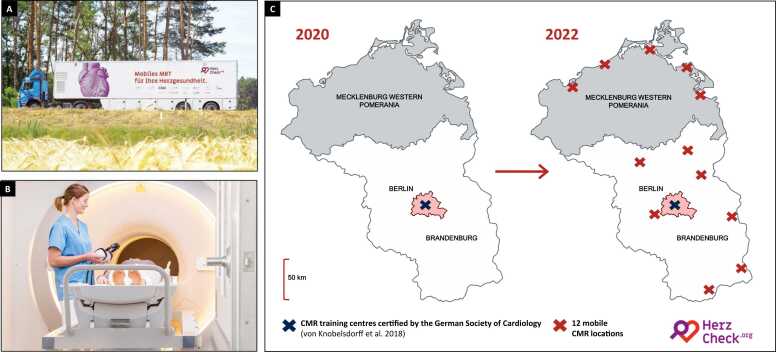
The mobile HERZCHECK screening units (A) are equipped with a state-of-the-art, ready-to-use cardiovascular magnetic resonance (CMR) scanner (Philips, Amsterdam, The Netherlands), (B) and for the purpose of conducting routine blood draws and measuring vital signs. (C) Whereas centers certified to offer training in CMR were only available in the German capital Berlin at the time of study initiation, HERZCHECK established high-quality CMR diagnostics at 12 sites in otherwise underserved rural regions of North-Eastern Germany. All 12 screening sites are served using mobile screening units operating under telemedical supervision from the Deutsches Herzzentrum der Charité in Berlin. Participants were referred for screening examinations at the established sites through >100 physicians who signed up for the HERZCHECK network or through a registry of individuals who are insured at one of the largest health insurance companies in North-Eastern Germany (AOK Nordost – Die Gesundheitskasse, Potsdam, Germany)

**Fig. 2:**
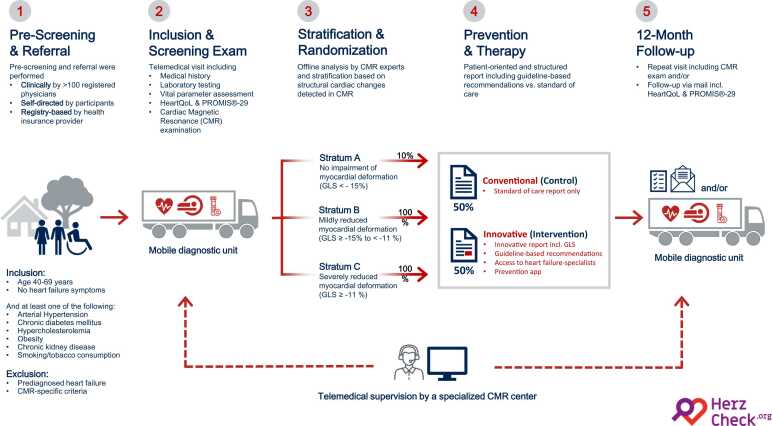
Overview of the HERZCHECK study design, including pre-screening, eligibility criteria, and enrollment, study examinations conducted at baseline, stratification, and randomization processes, and follow-up after 1 year. *BMI* body mass index, *CMR* cardiovascular magnetic resonance, *GLS* global longitudinal strain, *HF* heart failure

HERZCHECK is designed to evaluate the feasibility, diagnostic efficacy, and cost-effectiveness of a comprehensive, mobile, and telemedically-supervised diagnostic screening routine for asymptomatic pre-HF (stage B HF) in an at-risk population (stage A HF) in rural, structurally underserved regions of North-Eastern Germany. Specifically, HERZCHECK developed the infrastructural setup for the mobile screening units at 12 sites which were strategically chosen based on data gathered through a previously conducted questionnaire survey, showing that the majority of potential participants would be willing to travel 25–100 km (Fig. 1A-C) [11]. The central diagnostic methods employed in the assessed screening routine comprise a questionnaire-based medical history, laboratory testing, and a standardized, stress- and contrast-agent-free CMR exam. A visual summary of the HERZCHECK study design, including screening, enrollment, study examinations, randomization, and stratification procedures, is provided in Fig. 2.

### 2.2 Ethics

The HERZCHECK trial was approved by the Ethics Committee of Charité-Universitätsmedizin Berlin, Germany (Application: EA4/204/20) and registered at ClinicalTrials.gov (NCT05122793). HERZCHECK is conducted in accordance with the Declaration of Helsinki and relevant Guidelines for Good Clinical Practice. All participants provide written informed consent for participation in the study.

### 2.3 Study population, screening, and enrollment

HERZCHECK enrolls consecutive individuals, residing in the federal states of Brandenburg and Mecklenburg-Western Pomerania in Germany, based on the eligibility criteria summarized in Table 1. In brief, asymptomatic individuals between the age of 40 and 69 years without known HF, but with existing statutory health insurance and at least one of the following established cardiovascular risk factors—hypercholesterolemia, arterial hypertension, obesity, smoking/tobacco consumption, chronic diabetes mellitus, chronic kidney disease—are eligible.

**Table 1:** Summary of the eligibility criteria for participation in the HERZCHECK trial.

Eligibility criteria for HERZCHECK
Inclusion criteria
1. Between 40 and 69 years old 2. Female, male, or diverse gender 3. Existing statutory health insurance 4. At least one of the following cardiovascular risk factors: • Hypercholesterolemia (anamnestic; lipid-lowering pharmacotherapy) • Arterial hypertension (anamnestic; antihypertensive pharmacotherapy) • Obesity (body mass index ≥30 kg/m^2^) • Smoking/tobacco consumption (anamnestic, past or present) • Chronic diabetes mellitus (anamnestic; antidiabetic pharmacotherapy) • Chronic kidney disease (CKD) (anamnestic CKD or eGFR ≤ 60 mL/min/1.73 m^2^) 5. Access to a smartphone 6. Ability to comprehend the nature and aims of the study, as well as the potential risk associated with participation 7. Ability to provide written informed consent for study participation and data processing
Exclusion criteria
1. Inability to provide informed consent 2. Previously diagnosed heart failure 3. General CMR exclusion criteria (pacemaker, defibrillator, intracranial aneurysm clips, metallic debris in the eyes) 4. Any other factors considered as exclusion criteria for a CMR exam by the investigators 5. Hemodynamic instability (heart rate <45/min, systolic blood pressure <90 mmHg) 6. Claustrophobia 7. Sensorineural hearing loss ≥30 dB or tinnitus 8. Acute mental disorders requiring therapy 9. (Potential) Pregnancy

*CMR* cardiovascular magnetic resonance*, dB* decibel*, eGFR* estimated glomerular filtration rate*, kg* kilograms*, m* meters*, min* minutes*, mL* milliliter*, mmHg* millimeters of Mercury

Key exclusion criteria include previously diagnosed HF; any contraindications for CMR (e.g., pacemaker, defibrillator, intracranial aneurysm clips, metallic debris in the eyes); hemodynamic instability (heart rate <45/min or systolic blood pressure <90 mmHg); claustrophobia; sensorineural hearing loss ≥30 dB or tinnitus; acute mental disorders requiring therapy; potential pregnancy; inability to provide informed consent.

Individuals are pre-screened and referred for evaluation of participation in the HERZCHECK trial by one of >100 physicians who signed up for the HERZCHECK network or through a registry of individuals who are insured at one of the largest health insurance companies in North-Eastern Germany (AOK Nordost – Die Gesundheitskasse, Potsdam, Germany). All individuals referred for evaluation of participation subsequently undergo a detailed telemedical screening on the day of the baseline visit. Only those individuals deemed eligible by the investigators are enrolled in the HERZCHECK trial.

### 2.4 Study examinations

All participants undergo a comprehensive diagnostic routine, including medical history taking, laboratory testing, CMR, and validated QoL questionnaires at baseline. As previously indicated in Fig. 2 and outlined in more detail in the section “follow-up,” the same diagnostic routine is repeated in a subset of participants after 12 months of follow-up. A patient-friendly video that was created to inform study participants about the on-site study procedures and examinations is provided as Supplementary Material S2.

#### 2.4.1 Medical history

A focused medical history of the participants and their close biological relatives is collected during a telemedical consultation with physicians of the study center. All information is gathered and documented using two structured questionnaires. Central elements include the presence of cardiovascular risk factors, signs, and symptoms associated with cardiovascular diseases, and previous cardiovascular hospitalizations.

#### 2.4.2 Laboratory testing

Unless declined by the participant, trained personnel at the examination sites perform a venous blood draw to fill one 3.5 mL serum and two 2 mL ethylenediaminetetraacetic acid blood collection tubes. Additionally, participants are asked to provide a urine sample. Both blood and urine samples are transferred to a central reference laboratory (IMD Labor Oderland GmbH, Frankfurt Oder, Germany) for analysis at the end of each day. A complete list of the blood and urine parameters collected can be found in Table 2.

**Table 2:** Selection of the relevant parameters assessed during the study visits in the HERZCHECK trial.

		Relevant parameters
Questionnaire-based anamnesis		History of cardiovascular risk factors (hypercholesterolemia, arterial hypertension, smoking/tobacco consumption, chronic diabetes mellitus, chronic kidney disease)
		History of CV disease (heart failure, coronary artery disease, myocardial infarction, arrhythmia, stroke)
		Previous cardiovascular hospitalizations
		CV-related symptoms (dyspnea, edema, chest pain, syncope)
		Current medication
		Exercise (frequency, intensity)
		Alcohol consumption (frequency)
		Previous cardiovascular hospitalizations
		COVID-19 vaccinations and/or infections
		Previous operations
		History of cancer
		Contraindications for CMR
Vital parameters		Blood pressure
		Heart rate
		Height
		Weight
Laboratory analysis	Blood	N-terminal pro B-type natriuretic peptide
		Iron, ferritin, transferrin, transferrin-saturation
		Creatinine, estimated glomerular filtration rate
		Total cholesterol, high-density lipoprotein and low-density lipoprotein cholesterol, triglycerides
		Hemoglobin A1c
		Complete blood count without differential
		Sodium, potassium, chloride
	Urine	Creatinine
		Albumin
Cardiovascular magnetic resonance imaging	Left ventricle	Global longitudinal strain
		Ejection fraction
		End-diastolic diameter
		End-diastolic volume
		End-systolic volume
		Stroke volume
		Cardiac output
		End-diastolic thickness of the basal septum and lateral wall
		Myocardial mass
	Right ventricle	Ejection fraction
		End-diastolic diameter
		End-diastolic volume
		End-systolic volume
		Stroke volume
	Atria	End-diastolic diameter
	Mapping (contrast-free)	T1 relaxation time
		T2 relaxation time
	Ascending Aorta	Diameter
Quality of life questionnaires		HeartQoL
		Promis®-29 profile

*CMR* cardiovascular magnetic resonance*, CV* cardiovascular*, QoL* quality of life

#### 2.4.3 Quality of life questionnaires

Self-administered versions of the heart disease–specific health-related “HeartQoL” questionnaire developed by the European Society of Cardiology and the generic health–related “PROMIS®-29 Profile” questionnaire, both of which have previously been validated, are distributed to assess participants’ QoL [12], [13].

#### 2.4.4 Cardiovascular magnetic resonance imaging and image analysis

**Fig. 3:**
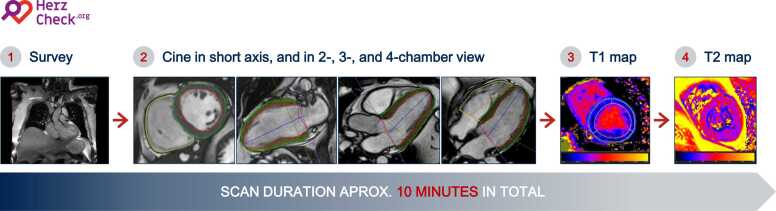
The CMR protocol used in HERZCHECK is designed for a comprehensive yet fast and concise assessment of cardiac structure and function that can be performed without the need for a physician on-site as no contrast- or stress-agents are administered. It includes a survey in three dimensions, cine imaging of the short- and long-axis views, as well as native T1 and T2 mapping. The total scan time is around 10 min. *CMR* cardiovascular magnetic resonance

All participants undergo a standardized CMR examination planned according to current recommendations of the Society of Cardiovascular Magnetic Resonance (SCMR) and performed by an extensively trained CMR technician in a fully mobile CMR unit [14]. The entire protocol (summarized in Fig. 3) is designed to take approximately 10 min and includes (a) a survey in three dimensions, based on which consecutive images are planned, (b) short-axis, two-, three- and four-chamber cine sequences, used to assess myocardial structure and function, and (c) T1- and T2-mapping sequences, used for myocardial tissue characterization. No contrast agents or pharmacological stress stimuli are administered during the CMR exam, omitting the need for a physician being present on-site. A comprehensive description of the image acquisition procedures is provided in Supplementary Material S3. Acquired images are sent to the study center where they are analyzed offline and in accordance with current consensus recommendations for the standardized image interpretation and post processing in CMR using cvi42® version 5.13.7 (Circle Cardiovascular Imaging Inc., Calgary, Alberta, Canada) [15]. All study investigators and CMR technicians were trained at the CMR-Academy of the German Heart Centre Berlin (CMR-Academy, Deutsches Herzzentrum Berlin, Berlin, Germany). All study investigators are certified according to the regulations of the SCMR. Quality assurance of the acquired CMR scans is carried out by a trained CMR technician, who provides immediate feedback and correction in the event of non-diagnostic image quality of individual scans. A detailed description of the image analysis procedures and a list of the most relevant analyzed CMR parameters may be found in Supplementary Material S4 and Table 2, respectively. The left ventricular global longitudinal strain (GLS), an index of myocardial deformation that is used as the central imaging parameter to assess myocardial dysfunction in the HERZCHECK trial, is quantified using a feature-tracking approach. RadioReport® version 0.32.3 (Neo Q Quality in Imaging GmbH, Berlin, Germany) is used to ensure structured reporting of all CMR exams.

### 2.5 Stratification

Previously derived cutoffs of the left ventricular GLS are used to stratify participants into those at-risk but without myocardial deformation impairment and those with myocardial deformation impairment [16]. Specifically, participants without myocardial deformation impairment are assigned to stratum A (GLS < −15%), participants with mild myocardial deformation impairment are assigned to stratum B (GLS ≥ −15% to < −11%), and participants with severe myocardial deformation impairment are assigned to stratum C (GLS ≥ −11%). According to the 2022 AHA guidelines for the management of HF, all participants with a reduced GLS (strata B and C) are considered as having asymptomatic pre-HF (stage B HF) by the criterion of reduced left ventricular systolic function [6].

### 2.6 Randomization to a preventive strategy

Following CMR-based stratification, 10% of participants in stratum A, and all participants in strata B and C are randomized into two intervention arms—a conventional (control) group and an innovative (intervention) group—in a 1:1 ratio. The 10% of participants from stratum A comprise a random sample selected based on a predetermined quota using a computer-generated mechanism. To achieve balanced group sizes within the strata, a stratified block randomization approach is used. The randomization list is provided by the Department of Medical Statistics, University Medical Center Göttingen, Germany.

### 2.7 Trial interventions

#### 2.7.1 Conventional (control) group

Participants randomized to the conventional (control) group receive CMR and laboratory test reports, as well as a summarizing medical report aligned with the current standard of care (SoC). Except for information regarding the GLS, the SoC reports contain all numerical measurement results obtained from the CMR exam and laboratory tests. Additionally, they provide a cumulative summary and interpretation of the findings, including explicit reference to any observations considered pathological or requiring further diagnostic evaluation.

#### 2.7.2 Innovative (intervention) group

Beyond all information provided as SoC, participants randomized to the innovative (intervention) group receive reports containing information regarding their left ventricular GLS, as well as detailed and patient-friendly explanations of all findings from the CMR exam and laboratory tests. Furthermore, the innovative CMR test report and summarizing medical report contain recommendations for guideline-compliant pharmacotherapy, risk factor modifications, and lifestyle modifications for participants in strata B and C, as well as recommendations for further diagnostics for participants in stratum C [17], [18], [19], [20].

Participants in the innovative (intervention) group are also offered access to the password-protected “CardioCoach” prevention app (Bundesverband Niedergelassener Kardiologen Service GmbH, Munich, Germany) through their mobile phone or tablet. The “CardioCoach” app allows users to continuously document and monitor their measured vital parameters and promotes secondary prevention by providing detailed information on cardiovascular risk factors and ways to minimize them, such as exercise activities or a healthy diet.

In addition, the treating physicians of participants in the innovative group have the option of consulting a certified HF competence center (Heart and Vascular Center Bad Bevensen, Germany) via telemedicine for all questions regarding test results, further diagnostics, guideline-based pharmacotherapy, and preventive lifestyle changes.

### 2.8 Follow-up

All randomized participants (i.e., 10% of stratum A, and all of strata B and C) are followed for 12 months. Specifically, all randomized participants are asked to return completed versions of the “HeartQoL” and “PROMIS®-29 Profile” QoL questionnaires which they received by mail after 3, 6, 9, and 12 months.

In addition to these questionnaires, a subset of all randomized participants is asked to partake in a comprehensive follow-up examination 12 (±2) months after their baseline visit. The follow-up examination includes all study components also carried out at baseline (i.e., structured medical history, laboratory testing, CMR, QoL questionnaires).

All randomized participants who are not invited for or do not attend the above-described follow-up examination after 12 months are asked to return completed versions of the questionnaires used for the structured medical history, which are sent to them by mail.

### 2.9 Study endpoints

**Fig. 4:**
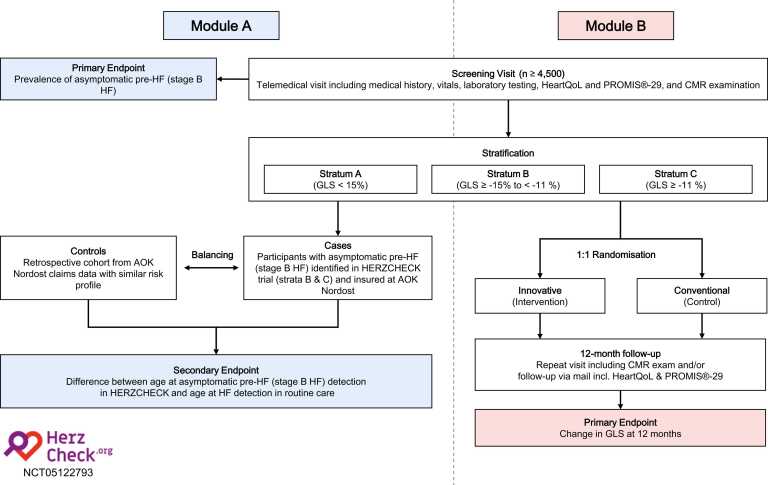
Overview of endpoints in the HERZCHECK trial. Module A is a cross-sectional study to determine the primary endpoint of the prevalence of unrecognized asymptomatic pre-HF (i.e., stage B HF) in a rural at-risk (i.e., stage A HF) population. The secondary endpoint of module A is the mean difference between the age at asymptomatic pre-HF (stage B HF) detection in the HERZCHECK program and the age at HF detection in the absence of a structured screening mechanism (routine care). Module B is a subsequent CMR–assessed randomized controlled trial following a “Prospective Randomized Open Blinded Endpoint” (PROBE) design, which aims to evaluate the efficacy of innovative prevention approaches in a prespecified subset of participants from module A. *CMR* cardiovascular magnetic resonance, *HF* heart failure, *GLS* global longitudinal strain

HERZCHECK is designed as a trial consisting of two modules, each with distinct endpoints. Module A is a cross-sectional study to determine the prevalence of unrecognized asymptomatic pre-HF (stage B HF) in a rural at-risk population. Module B is a subsequent CMR–assessed randomized controlled trial following a PROBE design, which aims to evaluate the efficacy of innovative prevention approaches in a prespecified subset of participants from module A. An overview of study endpoints is given in Fig. 4.

The primary endpoint for module A is a CMR-derived GLS ≥ −15%, which is equivalent to the diagnosis of asymptomatic pre-HF (stage B HF) by the criterion of reduced left ventricular systolic function according to the 2022 AHA HF guidelines [6]. The secondary endpoint of module A is the mean difference between the age at asymptomatic pre-HF (stage B HF) detection in the HERZCHECK program and the age at HF detection in the absence of a structured screening mechanism (routine care).

The primary endpoint for module B is the change in CMR-derived GLS between baseline and 12 months and is only assessed in randomized participants attending the 12-month follow-up examination. Secondary endpoints for module B are assessed in all participants who underwent randomization and include the occurrence of adverse cardiac events, as well as changes in blood parameters and participant-reported outcomes (e.g., QoL) between baseline and 12 months. Additionally, module B includes a health economic evaluation of the proposed HERZCHECK screening routine.

### 2.10 Statistical considerations

#### 2.10.1 Sample size calculation

Individual sample size calculations for modules A and B of the HERZCHECK trial were carried out using G*Power version 3.1.9.7 (https://stats.oarc.ucla.edu/other/gpower/).

For module A, the sample size was initially calculated such that the prevalence of unrecognized asymptomatic pre-HF (stage B HF; by the criterion of a GLS ≥ −15%) in the prespecified population at-risk (stage A HF) can be determined with an accuracy (half width of the 95% confidence interval) of ±0.5% at an assumed prevalence of 5% or, equivalently, ±1.0% at an assumed prevalence of 20%. These initial calculations resulted in a sample size of 6600 participants required to undergo the baseline CMR examination. Due to the SARS-CoV-2 pandemic, recruitment was initially slower than expected. Therefore, the Steering Committee decided to recalculate the sample size based on the observed prevalence of unrecognized asymptomatic pre-HF (stage B HF; by the criterion of a GLS ≥ −15%). Specifically, the revised calculations resulted in a required sample size of 4500 participants.

For the evaluation of module B, the population is considered by GLS-stratum with strata A, B, and C expected to contribute approximately 75%, 20%, and 5% to the study population, respectively. As stratum C is expected to be the smallest and associated with the highest risk, sample size calculations for module B focus on this stratum as follows. To demonstrate a clinically relevant difference in the primary endpoint (i.e., change in GLS between baseline and 12 months) between the conventional (control) and innovative (intervention) groups, a test for differences in means at the two-sided significance level of 5% is applied. Clinical relevance was assumed at a difference in ΔGLS ≥ 1% between groups with a within-group standard deviation of 2.9% [21], [22], [23]. With a total of 330 participants (6600 × 0.05) in stratum C expected to be randomized to the two study groups at a ratio of 1:1 and adjusting for an expected dropout rate of 20%, this test achieves a power of 80%. As the remaining risk strata (i.e., A and B) are considerably larger, smaller effect sizes are also expected to lead to a power of 80%. For stratum A (after random sampling) and B, a total of 496 (6600 × 0.75 × 0.1) and 1320 (6600 × 0.2) participants are expected to be randomized, respectively. Consequently, the total sample size in module B adds up to 2146 participants (1320 + 496 + 330), allocated at a 1:1 ratio to both study groups.

As a result of the sample size recalculation, strata B and C were merged for the evaluation of module B based on the classification of asymptomatic pre-HF (stage B HF) by the AHA [6]. Merging strata B and C led to updated planning assumptions (as of December 2022), as stratum B is associated with a lower risk than stratum C and, as a result, differences in ΔGLS are expected to be smaller. Specifically, clinical relevance was assumed at a difference in ΔGLS ≥ 0.85% between study groups. The within-group standard deviation was adjusted to 2%. Under these considerations, the comparison of the primary endpoint in module B achieves a power of 83%, including a total of 244 patients after dropout adjustment.

#### 2.10.2 Statistical analysis

Statistical analyses for the primary endpoint of module A include all participants who are enrolled in the study and complete the baseline examination. Participants’ baseline characteristics are reported using appropriate descriptive statistics and graphical methods. The prevalence of asymptomatic pre-HF (stage B HF) by the criterion of reduced left ventricular systolic function (i.e., GLS ≥ −15%) is estimated by calculating the proportion of participants classified as stratum B or C [6], [24]. If not stated otherwise, tests are conducted two-sided at a 5% significance level. Effect sizes are reported with corresponding two-sided 95% confidence intervals.

For the evaluation of the secondary endpoint of module A, the mean difference between the age at detection of asymptomatic pre-HF (stage B HF) in the HERZCHECK trial and the age at HF detection in routine care is estimated by comparing HERZCHECK trial data of participants in strata B and C to those of a historical control group constructed from claims data of one of the largest statutory health insurance companies in North-Eastern Germany (AOK Nordost – Die Gesundheitskasse, Potsdam, Germany). This control group is selected to have a comparable risk profile to the study participants in strata B and C by covariate balancing and is followed for 9 years to identify incident HF cases [25], [26], [27]. The robustness of the results is tested by sensitivity analyses, and subgroup analyses might be conducted according to, for example, sex and asymptomatic pre-HF severity (stratum).

Concerning module B, the primary endpoint (i.e., change in GLS from baseline to month 12) is evaluated using analysis of covariance including baseline GLS as well as risk stratum and intervention with corresponding interaction as explaining variables. The primary evaluation follows the intention-to-treat principle. Missing values are dealt with using appropriate multiple imputation methods [28]. Analyses of secondary endpoints, including laboratory parameters, QoL, and adverse cardiac events, are conducted to compare the two study arms, stratified by risk factors where appropriate. Additionally, conventional risk markers are correlated with GLS.

A health economic evaluation is conducted from the perspective of the German statutory health insurance. First, using a decision-analytical model, a long-term cost-effectiveness analysis is conducted [29]. Comparing the lifetime costs and clinical outcomes of the HERZCHECK program and routine care without screening and prevention, the incremental cost-effectiveness ratio is calculated (e.g., costs per life year gained). Second, a short-term cost analysis for participants without identified asymptomatic pre-HF (stratum A) is conducted over the 12-month study period. Overall costs and costs in different categories (e.g., outpatient care, inpatient care, medication) are compared between the conventional (control) and innovative (intervention) groups. The robustness of the results is analyzed by different sensitivity analyses [30], [31]. Subgroup analyses might be conducted to evaluate differences in results based on, for example, sex or asymptomatic pre-HF severity (stratum). Data are obtained from HERZCHECK trial data, as well as claims data, and are supported by literature where necessary.

If not otherwise specified, data preparation and statistical analyses are performed using R version 4.2.2 or higher (R Foundation for Statistical Computing, Vienna, Austria) and IBM SPSS Statistics for Windows version 29.0.0.0 or higher (IBM Corporation, Armonk, New York). Additionally, TreeAge Pro Healthcare version 2023 (TreeAge Software LLC, Williamstown, Massachusetts) is used for health economic evaluations.

## 3 Discussion

With the aim of improving early detection and providing an opportunity for timely interventions that delay or prevent the progression to manifest HF, HERZCHECK is the first large-scale study to evaluate a comprehensive screening approach for asymptomatic pre-HF (stage B HF). To maximize its potential impact, HERZCHECK focuses on individuals aged 40–69 years with at least one established cardiovascular risk factor, representing a well-defined at-risk population (in line with AHA stage A HF) young enough for the additionally evaluated prevention interventions to have a substantial long-term impact [6].

To date, the detection of biomarker changes by means of natriuretic peptide measurements constitutes the only guideline-recommended screening approach for asymptomatic pre-HF (stage B HF) [6]. The screening algorithm investigated in HERZCHECK incorporates this existing approach alongside a structured medical history, but expands it to include state-of-the-art cardiac imaging, which enables the detection of structural heart disease as an additional key aspect of stage B HF [6]. Special emphasis is placed on the GLS, an index of myocardial deformation that has been shown to detect early changes in HF more accurately than conventional imaging parameters (e.g., left ventricular ejection fraction) [32], [33], [34]. Although also quantifiable by echocardiography, HERZCHECK uses a concise, stress- and contrast-free CMR examination for cardiac imaging. This is mainly because CMR is more reproducible, less observer-dependent, and reliably feasible in obese individuals, who make up a significant proportion of the studied population, but in whom echocardiography fails to provide diagnostic image quality in more than 20% of cases. [35], [36].

Previous epidemiological studies have demonstrated strong regional variations in the prevalence of HF, with rural areas showing a 40% higher adjusted risk than urban ones [10]. This disparity is further aggravated by shortages of qualified and specialized health care professionals in rural areas [9]. To aid in finding solutions to this growing problem, the HERZCHECK screening is specifically tailored to the needs of and tested in structurally weak rural regions. Encouraged by a previous study that demonstrated the feasibility of mobile CMR exams, HERZCHECK, thus, developed and now uses the required infrastructure for fully mobile screening units centered around a modern CMR device and operating under telemedical supervision from one of Europe's leading cardiology centers (Deutsches Herzzentrum der Charité, Berlin, Germany) (Figs. 1A-C and 2) [37]. This approach enables the demand-oriented provision of high-tech diagnostics without the need for specialized physicians to be on-site, while ensuring the highest medical and safety standards.

Apart from performing a comprehensive evaluation of the proposed screening algorithm, the primary research interest of HERZCHECK is to clarify previous estimates of the prevalence of asymptomatic pre-HF (stage B HF) in the at-risk population, which ranges from less than 15% to more than 40% in different studies, and to improve our understanding of the influence of individual risk factors on the development and progression of HF [38], [39], [40], [41]. However, through its holistic approach, focusing not only on the heart but several other associated organ systems, HERZCHECK is also intended to serve as an extensive database of validated medical information, which could find far-reaching applications in future medical care planning for structurally weak areas and in optimizing interdisciplinary disease prevention.

## 4 Conclusion

In essence, HERZCHECK provides a blueprint for a comprehensive, contemporary screening approach tailored to the needs of the targeted population. By implementing this approach in a representative cohort of approximately 4500 participants, HERZCHECK will provide important new information about (a) the prevalence of asymptomatic pre-HF (stage B HF) in a structurally underserved rural at-risk (stage A HF) population and (b) the feasibility, added diagnostic value, and health economic aspects of CMR exams as part of future screening mechanisms for HF in clinical routine care.

## Funding

The HERZCHECK trial was fully funded by the “Innovation Fund for the promotion of new forms of care” of the 10.13039/501100014840Federal Joint Committee (Gemeinsamer Bundesausschuss); Funding reference: 01NVF19014.

## Author contributions

Norbert Frey: Writing—review and editing, Resources, Conceptualization. Burkert Pieske: Writing—review and editing, Resources. Sebastian Kelle: Writing—review and editing, Writing—original draft, Visualization, Validation, Supervision, Software, Resources, Project administration, Methodology, Investigation, Funding acquisition, Formal analysis, Data curation, Conceptualization. Katrin Christiane Reber: Writing—review and editing, Resources, Project administration, Methodology, Investigation, Conceptualization. Ulf Landmesser: Writing—review and editing, Resources. Anna Clara Nolden: Writing—review and editing, Writing—original draft, Visualization, Validation, Supervision, Project administration, Methodology, Investigation, Formal analysis, Data curation. Maximilian Leo Müller: Writing—review and editing, Visualization, Validation, Supervision, Software, Project administration, Methodology, Investigation, Formal analysis, Data curation. Alexander Huppertz: Writing—review and editing, Software, Resources, Methodology, Investigation. Dusan Simic: Writing—review and editing, Validation, Supervision, Methodology, Formal analysis, Conceptualization. Matthias Issing: Writing—review and editing, Software, Resources, Project administration, Methodology, Funding acquisition, Conceptualization. André Glardon: Writing—review and editing, Software, Resources, Project administration, Methodology, Funding acquisition, Conceptualization. Dirk Müller: Writing—review and editing, Validation, Resources, Methodology, Formal analysis. Arim Shukri: Writing—review and editing, Validation, Formal analysis. Undine Ella Witt: Writing—review and editing, Validation, Software, Project administration, Methodology, Investigation, Formal analysis. Florian André: Writing—review and editing, Project administration, Methodology. Alex Tuit: Validation, Supervision, Software, Project administration, Methodology, Formal analysis, Data curation. Mina Cvetkovic: Writing—review and editing, Visualization, Validation, Software, Project administration, Investigation, Formal analysis. Sein Schmidt: Writing—review and editing, Resources, Methodology, Conceptualization. Volkmar Falk: Writing—review and editing, Resources, Project administration, Conceptualization. Tim Friede: Writing—review and editing, Validation, Supervision, Resources, Project administration, Methodology, Funding acquisition, Formal analysis, Conceptualization. Rebecca Elisabeth Beyer: Writing—review and editing, Writing—original draft, Visualization, Validation, Supervision, Software, Project administration, Methodology, Investigation, Formal analysis, Data curation. Stephanie Stock: Writing—review and editing, Validation, Supervision, Software, Resources, Project administration, Methodology, Formal analysis, Conceptualization. Johannes Wieditz: Writing—review and editing, Writing—original draft, Validation, Formal analysis, Data curation. Hannah Kentenich: Writing—review and editing, Writing—original draft, Validation, Formal analysis, Data curation. Gisela Thiede: Writing—review and editing, Writing—original draft, Validation, Supervision, Project administration, Methodology, Funding acquisition, Formal analysis, Data curation, Conceptualization. Henning Steen: Writing—review and editing, Project administration, Methodology, Funding acquisition, Conceptualization. Bjoern Andrew Remppis: Writing—review and editing, Writing—original draft, Supervision, Methodology, Funding acquisition, Formal analysis, Conceptualization.

## Declaration of generative AI and AI-assisted technologies in the writing process

No generative AI was used during the preparation of this article.

## Declaration of competing interests

S.K. reports grants and other support by DZHK (German Center for Cardiovascular Research), partner site Berlin, Philips Healthcare, Astra Zeneca and Myocardial Solutions outside of the submitted work. B.P. reports grants from the DZHK (German Center for Cardiovascular Research), partner site Berlin, and the DFG (German Research Foundation), Astra Zeneca, BMS, Novartis, Bayer Healthcare, and MSD outside the submitted work. All other authors declare that they have no relationships relevant to the contents of this paper to disclose.
