# Supplementary material for: Rationale and design of the HERZCHECK trial: Detection of early heart failure using telemedicine and cardiovascular magnetic resonance in structurally weak regions (NCT05122793)
Source: J Cardiovasc Magn Reson. 2025 Jan 15;27(1):101841. doi: 10.1016/j.jocmr.2025.101841 (PMC11870244; doi:10.1016/j.jocmr.2025.101841)

Supplementary Table S1: Members of the HERZCHECK consortium and technical partners involved in the project

Members of the HERZCHECK consortium			
Member	Role(s)	Responsible individual	Contact
**Deutsches Herzzentrum der Charité (DHZC)**	• Leader of consortium<br>• Study design<br>• (Tele-)Medical supervision of study examinations<br>• Data acquisition<br>• Oversight of data management and analysis	Prof. Dr. med. Sebastian Kelle	Augustenburger Platz 1, 13353 Berlin, Germany<br>https://www.dhzc.charite.de
**AOK Nordost – Die Gesundheitskasse**	• Pre-screening of eligible patients<br>• Provision of matched control cohort for module A<br>• Provision of routine health insurance data<br>• Intellectual input	Diana Graja	Brandenburger Straße 72, 14467 Potsdam, Germany<br>https://www.aok.de/pk/nordost/
**Herz- und Gefäßzentrum Bad Bevensen**	• Expert centre for telemedical consultation by treating physicians of patients randomized into the innovative (intervention) arm<br>• Intellectual input	Prof. Dr. med. Bjoern Andrew Remppis	Römstedter Straße 25, 29549 Bad Bevensen, Germany<br>https://www.hgz-bb.de

**Universitätsmedizin Göttingen**	• Case number calculations<br>• Medical statistics<br>• Data management<br>• Intellectual input	Prof. Dr. Tim Friede	Robert-Koch-Straße 40, 37075 Göttingen, Germany<br>https://www.umg.eu
**Universitätsklinik Köln**	• Health economic evaluation<br>• Intellectual input	Prof. Dr. med. Stephanie Stock	Kerpener Straße 62, 50937 Köln, Germany<br>https://www.uk-koeln.de
**Universitätsklinikum Heidelberg**	• Intellectual input	Prof. Dr. med. Norbert Frey	Im Neuenheimer Feld 672, 69120 Heidelberg, Germany<br>https://www.klinikum.uni-heidelberg.de
**medneo GmbH**	• Provision and technical operation of mobile screening units	PD Dr. med. Henning Steen	Reinhardtstraße 23-27, 10117 Berlin, Germany<br>https://www.medneo.com
Technical partners involved in HERZCHECK			
Member	Role	Responsible individual	Contact
**IMD Labor Oderland GmbH**	• Laboratory analyses of blood and urine samples	–	Franz-Mehring-Straße 23 A, 15230 Frankfurt (Oder), Germany<br>https://www.imd-oderland.de/de
**Neo Q Quality in Imaging GmbH**	• Provision of *RadioReport®* reporting software	Prof. Dr. med. Alexander Huppertz	Salzufer 15/16, In der Lanolinfabrik, 10587 Berlin, Germany<br>https://radioreport.com/de/home/

**Doc Cirrus GmbH**	• Provision of *inSuite* clinical information system	–	Pohlstraße 20, 10785 Berlin, Germany https://www.doc-cirrus.com
**Circle Cardiovascular Imaging Inc.**	• Provision of *CVI42®* cardiac imaging software	–	Suite 1100, 800 5th Avenue SW, Calgary, AB, Canada T2P 3T6 https://www.circlecvi.com
**mDAKS GmbH**	• Data export<br>• Technical support	Leo Rave	Seelachstrasse 3, 74177 Bad Friedrichshall, Germany https://www.mdaksgmbh.de
**Bundesverband Niedergelassener Kardiologen (BNK) Service GmbH**	• Provision of *CardioCoach* cardiovascular prevention app	–	Brabanter Straße 4, 80805 München, Germany https://www.bnk-cardiocoach.de/#/home
**cmr Akademie GmbH**	• Training of study investigators and CMR-technicians	Prof. Dr. med. Eckart Fleck	Augustenburger Platz 1, 13353 Berlin, Germany https://www.cmr-academy.com
**DHZB Foundation**	• Training of study investigators and CMR-technicians	Andreas Portmann	Amrumer Str. 32, 13353 Berlin, Germany https://dhzb.org/en

Supplementary Material S2

Online-video for patient information.

https://youtu.be/zGpePN2_s0Q?si=5_OcwabtolVrObsT

Image Acquisition

Standard Operating Procedure

General & Setup

- CMR data are acquired on a mobile 1.5T MRI system (Ingenia dStream, Philips Healthcare, Best, the Netherlands).
- A phased-array coil consisting of a flexible anterior- and a posterior part integrated in the patient bed is employed with up to 28 coil elements used for signal reception depending on the size and location of the acquired field of view.
- Height and weight are recorded at the beginning of the scan.
- ECG-gated scanning is performed in supine position.
- All images are planned and acquired according to the SCMR Standardized cardiovascular magnetic resonance imaging (CMR) protocols: 2020 update (1)

1. Kramer CM, Barkhausen J, Bucciarelli-Ducci C, Flamm SD, Kim RJ, Nagel E. Standardized cardiovascular magnetic resonance imaging (CMR) protocols: 2020 update. Journal of Cardiovascular Magnetic Resonance. 2020;22(1):17.

Localizers, RAO and p4CH

- Localizer images in the coronary, sagittal and transaxial axes are obtained.
- Subsquently RAO (right anterior obliques) and p4CH are planned on which all long axis and short axis cines and mapping images are planned.

CINE

- Cine images are acquired using a retrospectively gated balanced SSFP sequence with a repetition time (TR) = 3.30 ms, echo time (TE) = 1.65 ms, flip angle = 60°, acquired voxel size = 1.8 × 1.7 × 8.0 mm3 , twofold SENSE acceleration and 22 / 35 acquired / reconstructed phases per cardiac cycle. Cine images are acquired with multiple breath holds in two-chamber (2CH), three-chamber (3CH), and four-chamber (4CH) planes. Additionally, a full stack of short-axis (SAX) slices covering the entire LV with 14 slices (no slice gap) is acquired, with TR = 2.90 ms, TE = 1.45 ms, flip angle = 60°, acquired voxel size = 2.0 × 2.0 × 8.0 mm^3 and 20 / 35 acquired / reconstructed phases per cardiac cycle

Native T1 and T2 Mapping

- All T1 and T2 maps are obtained at least twice to minimize motion or breathing artifacts and to ensure consistent image quality.
- Native T1-mapping is performed using a modified Look-Locker (MOLLI) 5s(3s)3s-scheme. Typical imaging parameters are as follows: Acquired voxel size = 2.0 × 2.0 × 10 mm3, reconstructed voxel size = 0.5 × 0.5 × 10 mm3, balanced SSFP readout, flip angle = 35°, parallel imaging (SENSE) factor = 2 and effective inversion times between 150 and 3382 ms.
- T2-mapping is performed using a black-blood-prepared, navigator-gated, free-breathing hybrid gradient (echo planar imaging, EPI) and a spin-echo multi-echo sequence (GraSE), with the following typical imaging parameters: TR = 1 heartbeat, 9 echoes (TE1 = 15 ms, delta TE = 7.7 ms), FA 90°, parallel imaging (SENSE = 2), EPI factor = 7, black-blood prepulse and breath-hold (scan duration about 14 s).

Supplementary Material S4: Image Analysis Standard Operating Procedure

Image Analysis

Standard Operating Procedure

Table of Content

1. General & Setup p. 1
2. Measurements p. 2
3. Volumetry p. 3
4. Mapping p. 4
5. Strain p. 5

General & Setup

- All images are analyzed offline using the same protocol, according to the SCMR Standardized image interpretation and post-processing in cardiovascular magnetic resonance - 2020 update. (1)
- Circle CVI42 (Circle, Canada) is used for all post-processing.
- As described in the Standard Operating Procedure for Image Acquisition (Supp. 1), the following sequences are available for analysis:
    - Cine SAX
    - Cine 4ch
    - Cine 2ch
    - Cine 3ch
    - 2x T1 mapping SA
    - 2x T2 mapping SA

Schulz-Menger J, Bluemke DA, Bremerich J, Flamm SD, Fogel MA, Friedrich MG, et al. Standardized image interpretation and post-processing in cardiovascular magnetic resonance - 2020 update. Journal of Cardiovascular Magnetic Resonance. 2020;22(1):19.

Measurements

- The following parameters is measured in basal, enddiastolic Cine short axis slice:
    - LV enddiastolic diameter (LVEDD)
    - Interventricular septal diameter (IVSD)
    - LV posterior wall diameter (LVPWD)
- RV enddiastolic diameter is measured in the basal, enddiastolic 3 chamber view, perpendicular to the LV long axis.
- The following parameters is measured in the endsystolic 4 chamber view:
    - Left atrial diameter
    - Right atrial diameter
- Aortic diameter is assessed in transaxial localizer images at the level of pulmonary artery bifurcation.

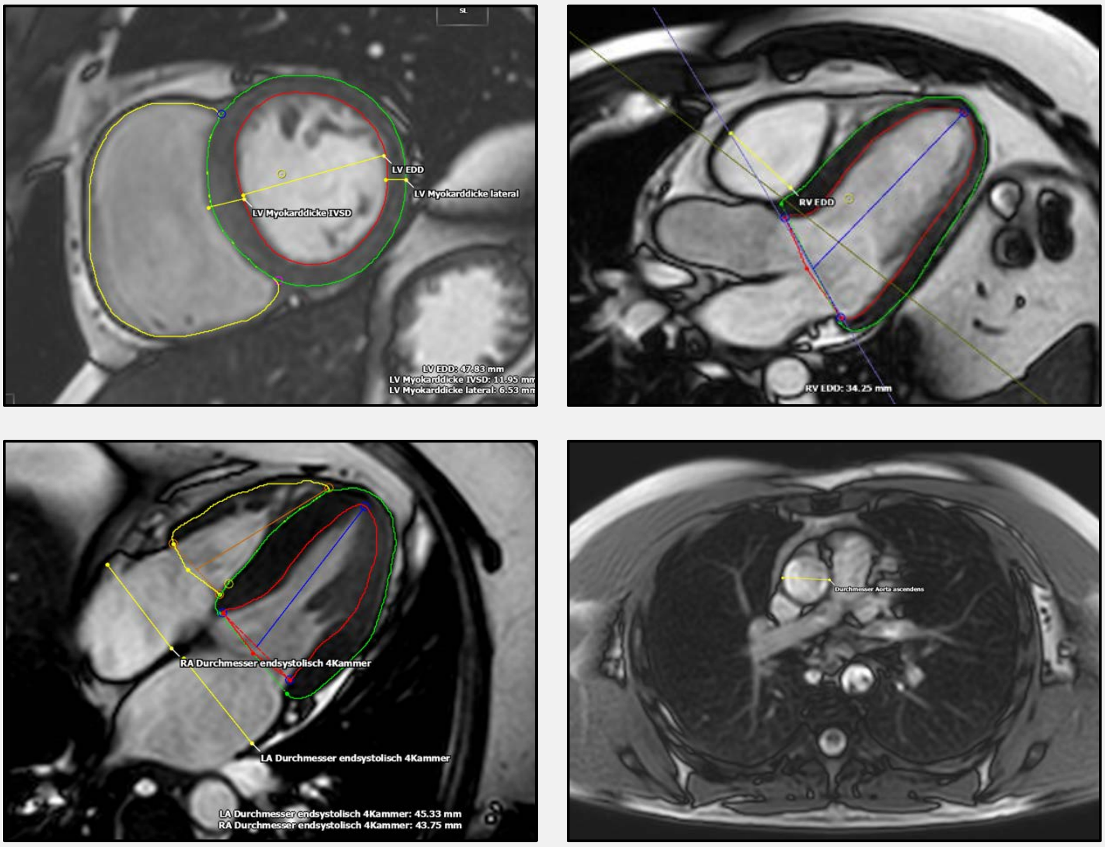

Volumetry

- For volumetry, enddiastole (ED) and endsystole (ES) are identified visually in accordance with the closure of respective valves.
- LV volume is assessed using the Simpson's method in short axis view. ED and ES contours are drawn along the anatomical borders in all slices of the short axis from apex to base. Basal slices with less than 50% myocardium are excluded.

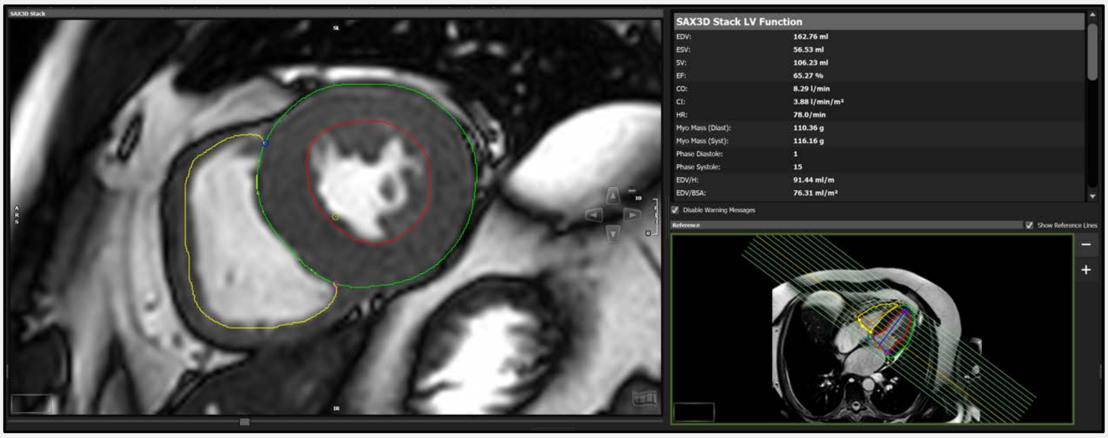

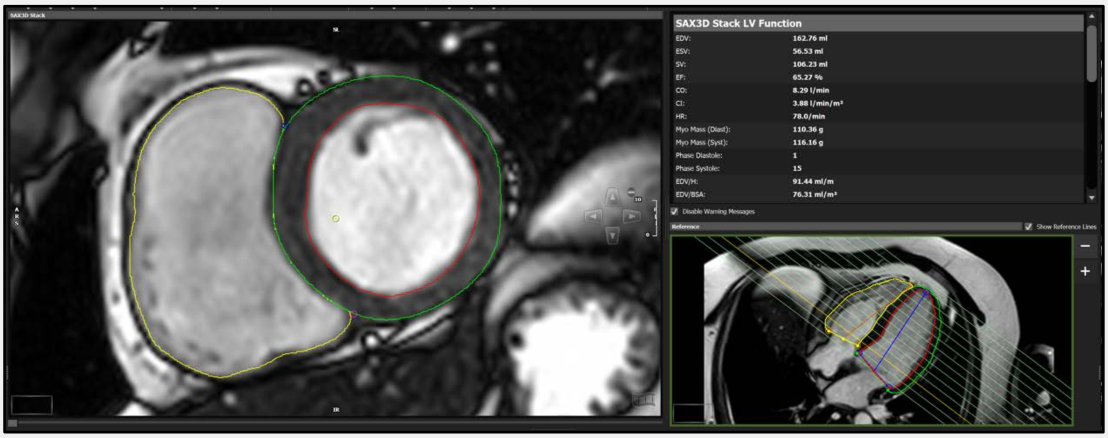

Mapping

- For analysis of native T1 maps and T2 maps, the respective image sequence of highest quality and with less signal to noise ratio or motion artifacts were selected from those available.
- Maps were automatically motion-corrected by the post-processing software.
- Mapping values were taken as average of values in the entire mid-ventricular septum, of which 15% of the outer and inner layer were automatically excluded by the software.
- In case of relevant artifact, a smaller region of interest, visually free of artifact, in the septum was selected.

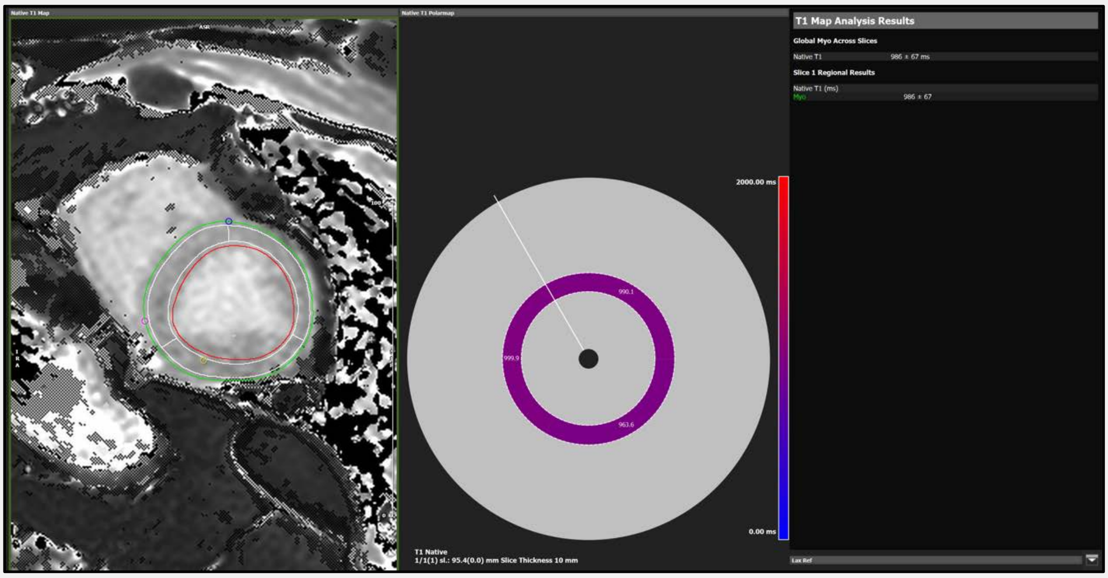

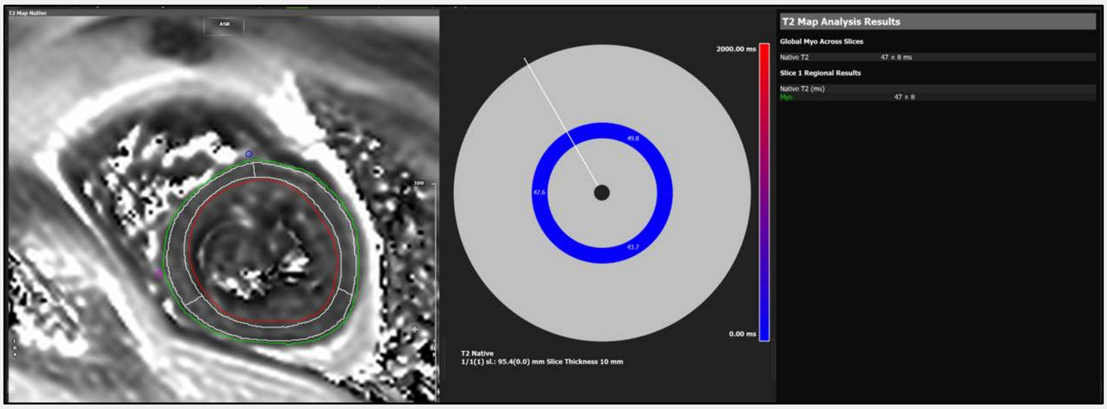

Strain

- Global longitudinal (GLS) and global circumferential strain (GCS) are assessed using feature-tracking in Cine long axis and short axis views respectively.
- From Cine short axis slices, any slices with incomplete myocardium or blood pool not depicted are excluded. From long axis slices, papillary muscles are carefully excluded.

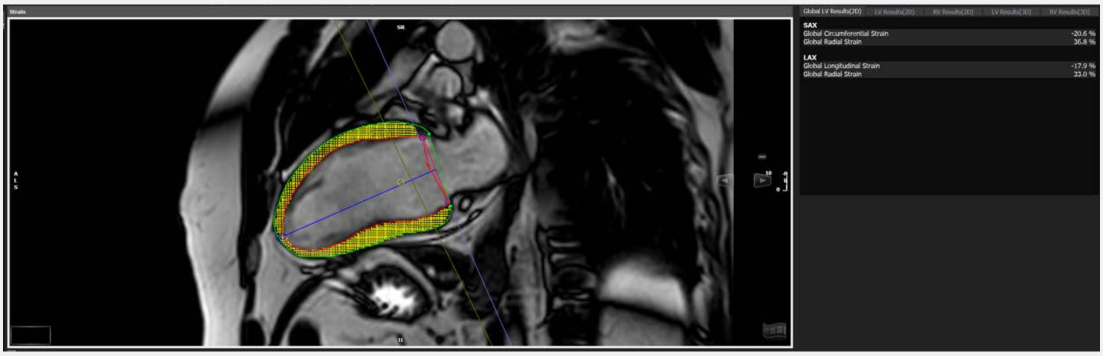

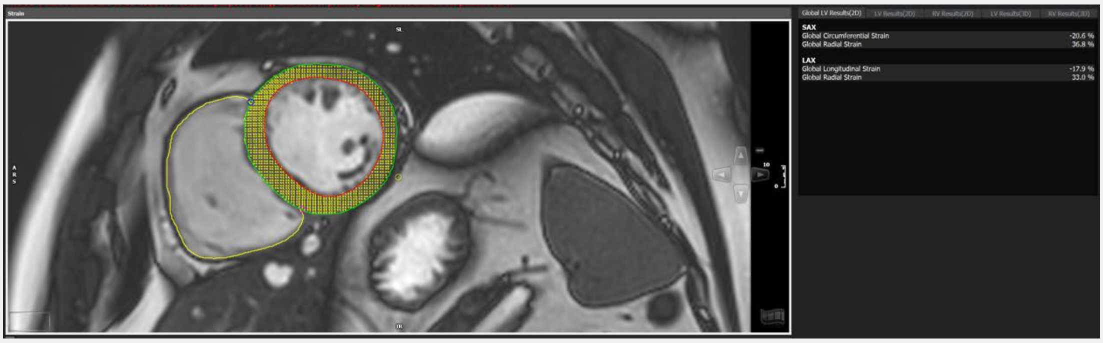

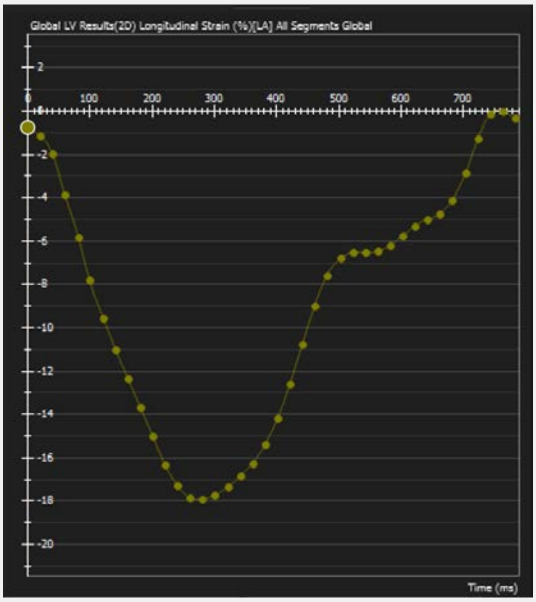

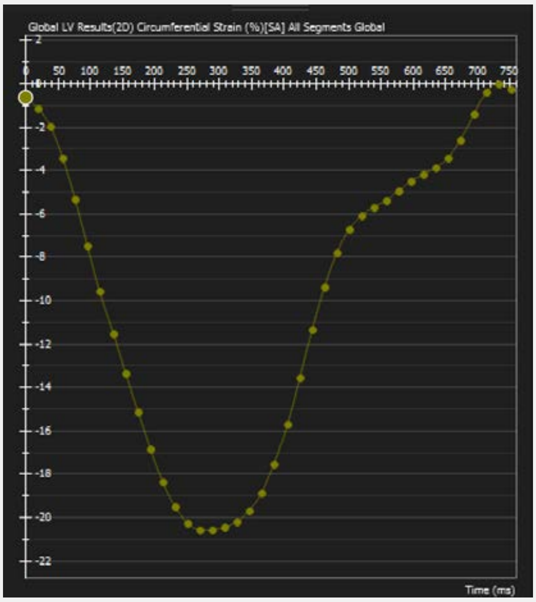

Supplement: Supplementary file 1 — Supplementary material [file mmc1.pdf]
